# Supplementary material for: Edible Far Eastern Ferns as a Dietary Source of Long-Chain Polyunsaturated Fatty Acids
Source: Foods. 2021 May 28;10(6):1220. doi: 10.3390/foods10061220 (PMC8228775; doi:10.3390/foods10061220)
Supplement: Supplementary file 1 [file foods-10-01220-s001.zip › foods-1211152-supplementary.pdf]

Supplementary Materials

Edible Far Eastern ferns as a dietary source of long-chain polyunsaturated fatty acids

Eduard V. Nekrasov, Vasily I. Svetashev

Table S1. Total lipids (TL, % of wet weight) and fatty acid content (% of sum) in the raw fiddleheads of edible ferns from the Russian Far East.

Table S2. Fatty acid content (mg/100 g wet weight) of raw fiddleheads of edible ferns from Amur Oblast, Russia.

Table S1. Total lipids (TL, % of wet weight) and fatty acid content (% of sum) in the raw fiddleheads of edible ferns from the Russian Far East.

	Pteridium aquilinum			*Matteuccia struthiopteris*			*Osmundastrum asiaticum*			Interspecies differences, P-value		
	Primorsky Krai	Amur Oblast	P-value	Primorsky Krai	Amur Oblast	P-value	Primorsky Krai	Amur Oblast	P-value	M.s. vs. O.a.	M.s. vs. Pt.a.	Pt.a. vs. O.a.
Total lipids, %	1.02±0.07	1.23±0.10	**0.049**	0.85±0.12	0.99±0.14	0.271	0.80±0.09	0.92±0.06	0.110	0.401	**0.027**	**0.004**
Fatty acid, %												
14:0	0.3±0.1	0.3±0.1	0.907	0.3±0.1	0.3±0.0	0.603	0.1±0.0	0.1±0.0	0.844	**0.001**	0.845	**0.000**
14:1n-5	0.2±0.0	0.1±0.0	0.060	0.1±0.0	0.2±0.0	**0.013**	0.2±0.0	0.1±0.0	**0.018**	0.337	0.991	0.204
15:0	0.1±0.0	0.1±0.0	0.623	0.3±0.0	0.2±0.0	**0.019**	0.1±0.0	0.1±0.0	0.494	**0.001**	**0.001**	**0.003**
16:0	26.3±0.9	25.0±0.8	0.133	26.6±0.6	26.6±1.0	0.927	30.0±0.3	25.3±0.5	**0.000**	0.384	0.117	0.131
16:1n-9	0.1±0.0	0.1±0.0	**0.032**	0.5±0.0	0.8±0.0	**0.000**	0.2±0.0	0.1±0.0	**0.035**	**0.001**	**0.001**	0.078
16:1n-7	0.2±0.0	0.3±0.1	0.097	0.9±0.0	1.2±0.1	**0.013**	0.2±0.0	0.2±0.0	0.165	**0.000**	**0.000**	0.224
16:1n-5	n.d.	0.1±0.0		n.d.	0.2±0.1		n.d.	0.2±0.0				
t-16:1n-13	0.1±0.1	0.2±0.1	0.595	0.2±0.0	0.3±0.1	0.139	0.2±0.1	0.1±0.0	**0.028**	0.093	0.062	0.868
16:2n-6	0.1±0.1	0.1±0.1	0.746	0.2±0.1	0.3±0.0	0.061	0.1±0.1	0.1±0.0	0.479	**0.035**	**0.040**	0.815
16:3n-3	1.7±0.2	1.9±0.6	0.693	1.3±0.1	1.7±0.4	0.310	2.5±0.3	2.8±0.2	0.141	**0.000**	0.167	**0.004**
17:0	0.1±0.0	0.1±0.0	0.064	0.1±0.0	0.2±0.0	0.068	0.1±0.0	0.1±0.0	0.180	0.064	**0.021**	0.205
18:0	1.7±0.0	2.0±0.2	0.076	1.0±0.0	1.5±0.1	**0.001**	1.6±0.5	1.2±0.0	0.273	0.697	**0.002**	**0.015**
18:1n-9	5.0±0.2	5.9±0.4	0.059	5.5±0.3	10.0±0.1	**0.001**	5.9±0.5	5.8±0.2	0.814	0.120	0.072	0.167
18:1n-7	0.6±0.0	0.9±0.2	0.117	1.5±0.0	1.6±0.2	0.485	1.0±0.1	1.0±0.3	0.949	**0.000**	**0.000**	0.055
18:1n-5	0.1±0.0	0.1±0.0	0.930	0.2±0.0	0.1±0.0	**0.002**	0.3±0.1	0.3±0.0	0.964	**0.011**	0.243	**0.000**
18:2n-6	27.0±0.4	25.1±0.8	**0.039**	20.9±0.5	19.9±0.7	0.143	18.0±1.6	17.0±1.0	0.414	**0.002**	**0.000**	**0.000**
18:3n-6	0.9±0.0	2.5±0.1	**0.002**	1.7±0.1	2.7±0.1	**0.001**	2.2±0.3	2.4±0.1	0.408	0.699	0.258	0.154
18:3n-3	10.8±0.6	12.8±1.3	0.094	16.9±0.1	14.4±1.1	0.062	22.0±1.3	24.0±1.2	0.119	**0.000**	**0.001**	**0.000**
18:4n-3	0.1±0.1	0.2±0.0	0.123	0.1±0.0	0.2±0.0	**0.048**	0.5±0.1	0.6±0.1	0.679	**0.000**	0.972	**0.000**
20:0	1.4±0.1	1.5±0.2	0.490	0.3±0.0	0.4±0.0	**0.015**	0.5±0.2	0.4±0.0	0.428	0.527	**0.000**	**0.000**
20:1n-9	0.1±0.0	0.1±0.0	0.080	0.2±0.0	0.2±0.0	**0.023**	0.1±0.0	0.1±0.0	0.306	**0.000**	**0.000**	0.761
20:2n-6	0.2±0.0	0.1±0.0	0.167	0.3±0.0	0.1±0.0	**0.001**	0.2±0.0	0.2±0.0	0.157	0.895	0.182	0.104
5,11,14-20:3	0.5±0.0	0.4±0.0	**0.009**	0.2±0.0	0.2±0.0	**0.006**	0.5±0.0	0.8±0.1	**0.021**	**0.001**	**0.002**	**0.017**

Table S1. *Cont.*

	Pteridium aquilinum			*Matteuccia struthiopteris*			*Osmundastrum asiaticum*			Interspecies differences, P-value		
	Primorsky Krai	Amur Oblast	P-value	Primorsky Krai	Amur Oblast	P-value	Primorsky Krai	Amur Oblast	P-value	M.s. vs. O.a.	M.s. vs. Pt.a.	Pt.a. vs. O.a.
Fatty acid, %												
20:3n-6	1.3±0.1	1.5±0.4	0.447	2.6±0.2	1.8±0.1	**0.018**	0.9±0.1	1.3±0.1	**0.022**	**0.001**	**0.005**	0.061
20:4n-6 (ARA)	13.5±0.4	11.8±0.4	**0.006**	12.8±0.5	9.3±0.1	**0.006**	6.4±0.5	8.8±0.2	**0.005**	**0.007**	0.114	**0.000**
20:3n-3	n.d.	0.03±0.00		n.d.	0.05±0.01		n.d.	0.1±0.0				
5,11,14,17-20:4	0.1±0.0	0.1±0.0	0.623	0.2±0.0	0.2±0.0	0.530	0.4±0.0	0.5±0.0	0.077	**0.000**	**0.000**	**0.000**
20:4n-3	0.1±0.0	0.1±0.0	0.193	0.2±0.0	0.1±0.0	0.454	0.2±0.0	0.3±0.0	**0.018**	**0.002**	**0.001**	**0.000**
20:5n-3 (EPA)	0.8±0.0	1.0±0.2	0.142	2.3±0.2	2.3±0.2	0.766	2.9±0.2	3.2±0.3	0.157	**0.001**	**0.000**	**0.000**
22:0	2.5±0.4	2.1±0.2	0.276	0.6±0.1	0.8±0.1	**0.010**	0.9±0.1	1.2±0.0	**0.043**	**0.002**	**0.000**	**0.000**
23:0	0.2±0.0	0.1±0.1	0.125	0.1±0.0	0.1±0.1	0.712	0.2±0.0	n.d.			0.354	
24:0	3.2±0.7	2.7±0.2	0.393	1.5±0.3	1.5±0.1	0.821	1.3±0.1	1.4±0.0	0.149	0.136	**0.001**	**0.001**
26:0	0.6±0.2	0.5±0.0	0.583	0.5±0.2	0.4±0.1	0.856	0.3±0.1	0.3±0.1	0.496	**0.027**	0.107	**0.001**
SFA	36.5±0.7	34.5±1.6	0.155	31.3±0.9	32.2±1.2	0.381	35.1±0.5	30.0±0.6	**0.000**	0.525	**0.001**	0.055
MUFA	6.4±0.1	7.8±0.3	**0.007**	9.1±0.3	14.6±0.2	**0.000**	8.1±0.4	7.9±0.2	0.466	**0.026**	**0.011**	**0.041**
PUFA	57.1±0.6	57.7±1.3	0.544	59.6±1.0	53.3±1.4	**0.005**	56.8±0.9	62.1±0.8	**0.002**	0.150	0.569	0.154
n-6	43.5±0.3	41.5±0.8	**0.037**	38.7±1.1	34.3±0.5	**0.013**	28.3±0.8	30.6±1.4	0.085	**0.000**	**0.001**	**0.000**
n-3	13.6±0.8	16.1±1.9	0.136	20.9±0.2	18.9±1.8	0.198	28.5±1.6	31.4±1.5	0.085	**0.000**	**0.001**	**0.000**
ARA/EPA	17.0	11.3		5.6	3.9		2.2	2.8				
(n-6)/(n-3)	3.2	2.6		1.8	1.8		1.0	1.0				

Abbreviations: ARA – arachidonic acid, EPA – eicosapentaenoic acid, M.s. – *Matteuccia struthiopteris*, MUFA – monounsaturated fatty acids, n.d. – not determined, O.a. – *Osmundastrum asiaticum*, Pt.a. – *Pteridium aquilinum*, PUFA – polyunsaturated fatty acids, SFA – saturated fatty acids.

Notes: Values are means of three samples ± standard deviations. P-values for difference between samples from the two regions are given next to the corresponding values. P-values for difference between species were calculated using the samples from the both regions. Statistically significant samples are highlighted in bold.

Table S2. Fatty acid content (mg/100 g wet weight) of raw fiddleheads of edible ferns from Amur Oblast, Russia.

Fatty acid	*Pteridium aquilinum*	*Matteuccia struthiopteris*	*Osmundastrum asiaticum*
14:0	2.1±0.5	1.5±0.2	0.6±0.0
14:1n-5	0.8±0.2	0.8±0.1	0.4±0.0
15:0	0.7±0.0	0.8±0.0	0.4±0.1
16:0	160.6±10.2	116.9±13.8	114.9±3.9
16:1n-9	1.0±0.1	3.4±0.5	0.6±0.0
16:1n-7	1.8±0.3	5.1±0.5	0.8±0.1
16:1n-5	0.4±0.1	0.9±0.6	0.9±0.1
t-16:1n-13	1.1±0.4	1.4±0.8	0.3±0.0
16:2n-6	0.8±0.3	1.4±0.2	0.5±0.0
17:0	0.9±0.1	0.7±0.0	0.7±0.0
16:3n-3	13.2±2.7	9.5±3.7	12.8±1.4
18:0	12.8±0.5	6.8±1.0	5.3±0.3
18:1n-9	38.1±4.0	45.0±5.8	26.4±2.0
18:1n-7	5.7±1.0	6.9±0.5	4.5±1.2
18:1n-5	0.7±0.2	0.3±0.2	1.3±0.1
18:2n-6	168.3±18.9	99.8±6.9	77.2±5.9
18:3n-6	16.9±1.0	14.5±1.8	10.9±0.8
18:3n-3	86.7±7.1	78.5±12.9	109.1±8.7
18:4n-3	1.3±0.1	1.0±0.1	2.5±0.3
20:0	9.2±1.7	1.9±0.3	1.6±0.0
20:1n-9	0.9±0.3	1.1±0.2	0.6±0.2
20:2n-6	0.9±0.1	0.7±0.1	1.0±0.1
5,11,14-20:3	2.3±0.2	1.0±0.1	3.6±0.3
20:3n-6	10.8±3.9	9.8±0.6	5.9±0.7
20:4n-6 (ARA)	80.1±6.5	52.5±3.3	40.2±2.8
20:3n-3	0.2±0.0	0.3±0.1	0.4±0.0
5,11,14,17-20:4	0.6±0.1	1.0±0.1	2.2±0.1
20:4n-3	0.8±0.2	0.8±0.1	1.4±0.1
20:5n-3 (EPA)	7.1±0.8	14.1±1.9	14.5±1.8
22:0	13.3±1.7	3.8±0.3	5.3±0.4
24:0	17.7±3.1	6.9±0.5	6.3±0.3
26:0	3.4±0.5	2.2±0.3	1.3±0.2
SFA	233.0±15.4	150.5±20.0	148.6±5.9
MUFA	50.4±3.7	65.0±8.9	35.8±1.0
PUFA	377.8±31.5	275.9±27.9	270.0±16.9
n-6	280.2±28.6	179.5±12.7	139.2±10.0
n-3	97.5±6.3	96.4±15.2	130.8±11.0
Total	661.2±49.7	491.4±56.8	454.3±23.8

Note: Values are means of three samples ± standard deviations. See Suppl. Table 1 for abbreviations.
